# Identification and prediction of patients eligible for augmented rehabilitation in emergency gastrointestinal surgery (RAUCisable): A protocol for a single-centre, retrospective, observational study

**DOI:** 10.1371/journal.pone.0350113

**Published:** 2026-06-03

**Authors:** Émilien Arnaud, Gilles Dequen, Émilie Chivé, Delphine Lignier, William Gacquer, Momar Diouf, Thibaut Balcaen, Daniel Aiham Ghazali, Jean-Marc Regimbeau

**Affiliations:** 1 Department of Emergency Medicine, Amiens-Picardy University Hospital, Amiens, France; 2 Modelling, Information and System lab (UR 4290), University of Picardy Jules Verne, Amiens, France; 3 Department of Gastrointestinal surgery, Amiens University Hospital, Amiens, France; 4 UR UPJV 7518 SSPC (Simplification of Care of Complex Surgical Patients) Research Unit, Jules Verne University of Picardie, Amiens, France; 5 Digital Services Department, Amiens-Picardy University Hospital, Amiens, France; 6 Department of Methodology, Biostatistics, Direction of Clinical Research, Amiens University Medical Hospital, Amiens, France; 7 Medical Information Department, Amiens-Picardy University Hospital, Amiens, France; 8 INSERM UMR1137, “Infection, Antimicrobials, Modelling, Evolution”, University of Paris-Diderot, Paris, France; Athens Medical Group, Psychiko Clinic, GREECE

## Abstract

**Introduction:**

Conditions requiring emergency gastrointestinal surgery pose substantial challenges to healthcare systems and patient outcomes. For emergency gastrointestinal surgery, the mortality rate is higher than after other types of surgery, and 30-day readmission rates can exceed 30%. Unlike elective surgery (for which the application of enhanced recovery after surgery (ERAS) procedures has led to demonstrably better recovery), emergency surgery patients are still managed in an ad hoc manner. The Réhabilitation Augmentée pour les Urgences Chirurgicales (RAUC) multi-faceted research program has been designed to transform the care of patients undergoing emergency gastrointestinal surgery [7]. The ancillary RAUCisable study will develop a classification model that can automatically flag up RAUC-eligible patients early in their visit to the emergency department (ED). The study’s secondary objectives include the identification of key features and the prediction of time to surgery.

**Method:**

RAUCisable is single-centre, retrospective, observational cohort study of electronic health records in the ED and digestive surgery department at Amiens-Picardie University Hospital (Amiens, France). All adult patients having attended the ED between January 1st, 2021, and December 31st, 2024, will be considered for inclusion. The primary classification outcome is eligibility for the RAUC pathway.

**Expected results:**

We expect to identify ~2,400 RAUC-eligible patients from among ~250,000–300,000 ED visits over a 4-year period. These patients are likely to be significantly older than non-surgical ED patients (e.g., more over-65s), with a higher proportion of acute abdominal conditions (e.g., ~ 24% with appendicitis, ~ 13% with bowel obstruction, ~ 10% with peritonitis, etc.), and greater disease acuity on triage.

**Discussion:**

The RAUCisable study’s findings will directly guide a concomitant, prospective, controlled study (RAUC-AMIENS) of the augmented recovery pathway, including ERAS elements and remote monitoring.

**Conclusion:**

The RAUCisable study is a pivotal step toward digitally enhanced emergency surgical care. By learning from past data, we are seeking to improve the future management of emergency surgery patients through timely identification and targeted care pathways. This protocol article details our methodological approach for ensuring rigor and reproducibility.

**Trial registration:**

NCT07037719

## Introduction

Conditions requiring emergency gastrointestinal surgery pose substantial challenges to healthcare systems and patient outcomes. In France alone, emergency departments (EDs) manage over 20.8 million visits per year [1]. Many of these visits involve patients who require emergency abdominal surgery, for which postoperative morbidity and mortality rates are significantly than for elective surgery [2]. Indeed, the mortality rate following reportedly up to five times higher for emergency gastrointestinal surgery than for planned elective procedures, and 30-day readmission rates can exceed 30% for major emergency abdominal surgery [3–5]. These poor outcomes are multifactorial and reflect patient frailty, the acute nature of the illness, and the lack of dedicated perioperative optimization protocols in emergency settings. In elective surgery scenarios, the application of enhanced recovery after surgery (ERAS) procedures have demonstrably improved recovery (*e.g.,* reducing complications, length of stay (LOS), and readmissions) [6], In contrast, emergency surgery patients are still managed in an *ad hoc* manner and lack an optimized, structured pathways. With a view to improving outcomes, there is a clear, unmet need for systematic, perioperative care pathways in emergency surgery. The multifaceted *Réhabilitation Augmentée pour les Urgences Chirurgicales* (RAUC) research programme has been designed to transform the care of patients undergoing emergency gastrointestinal surgery [7]. The RAUC project is funded as a hospital-university research initiative and represents a novel, comprehensive pathway for emergency surgical patients by adapting the ERAS principles (such as early mobilization, optimized analgesia, prompt postoperative feeding, and fewer drains and antibiotics) to emergency medicine and by leveraging digital health and artificial intelligence (AI) tools. Preliminary work by our group and others has suggested that applying ERAS principles to emergency medicine is feasible and can improve outcomes for patients with conditions such as appendicitis, cholecystitis [8,9], bowel obstruction, and peritonitis [10]. Furthermore, organizational optimization [11], e-health interventions (*e.g.,* remote monitoring and smart health devices) and machine learning algorithms have shown benefit in elective surgical care [12], for instance by enabling the earlier detection of complications [13]. However, these innovations have yet to be widely implemented in emergency surgical workflows.

Machine-learning applications are already being used in the ED – particularly for predicting patient outcomes (*i.e.,* admission to hospital or discharge to home) via the analysis of structured and/or unstructured datasets [14,15]

To address these gaps, the RAUC project comprises several interrelated studies:

1. RAUC-AMIENS [16], a clinical trial evaluating the effectiveness of an ERAS and e-health follow-up in emergency gastrointestinal surgery patients.
2. RAUC-IA, a study focusing on the development of an AI-driven triage algorithm using data from emergency and surgical care.
3. RAUC-OUVERT, a test of the RAUC pathway’s generalizability in another hospital.
4. RAUC-ALGO, a forthcoming study of the putative benefits of the AI triage algorithm in clinical practice.

During the implementation of these four interrelated studies, the need for a supplementary retrospective study – the RAUCisable study- emerged. Using routinely collected hospital data, the RAUCisable study will (i) identify patients who would have been eligible for the RAUC pathway and (ii) develop predictive models for the patients’ clinical course.

### Study rationale and significance

By retrospectively learning from four years of patient data, RAUCisable will provide the basis for an AI-driven triage algorithm that could assist emergency physicians with the early identification of surgical emergencies requiring activation of the RAUC pathway. RAUCisable will also provide evidence for refining the RAUC pathway and the design of the RAUC-AMIENS trial. For example, if the analysis identifies certain high-risk features (*e.g.,* advanced age, sepsis on admission, or specific imaging findings) that strongly predict poor outcomes, these can be targeted with enhanced interventions (*e.g.*, admission to the intensive care unit (ICU) or emergency surgery within six hours). Conversely, patients predicted to have uncomplicated courses could potentially benefit from accelerated pathways, including early discharge with remote monitoring.

The RAUCisable study’s findings will directly guide the implementation of AI-based triage in the prospective RAUC-ALGO study and may be generalizable to other centres seeking to improve their emergency surgery outcomes. By integrating modern digital tools into the clinical workflow, this project is in line with current efforts to harness AI for improved triage and care in the ED [17]. Herein, we present the full protocol for the RAUCisable study (in compliance with the SPIRIT [18] guidelines) and detail the objectives, methods, and analysis plan.

### Objectives

**Fig 1:**
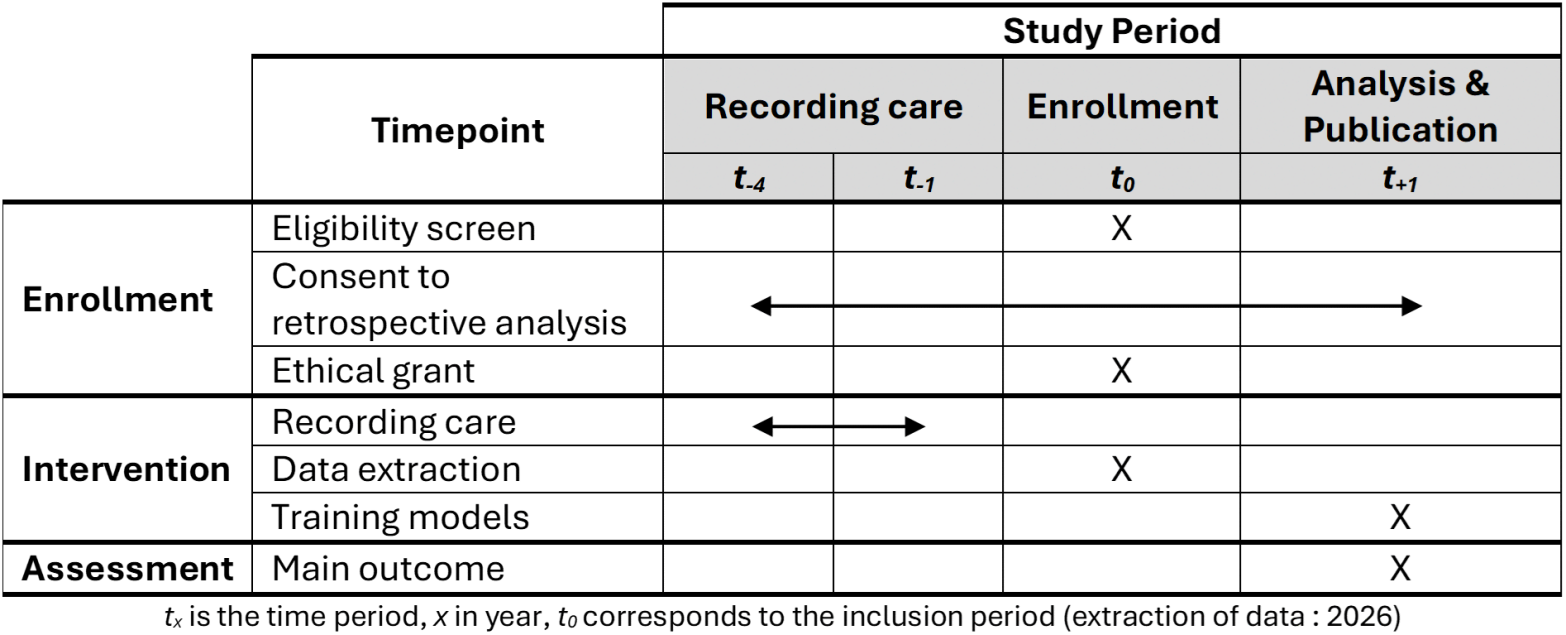
A schematic overview of the RAUC studies, including the RAUCisable study described here.

The primary objective of the RAUCisable study is to develop a binary classification model that can automatically flag up RAUC-eligible patients (RAUC + , in Fig 1) early in their visit to the ED.

The RAUCisable study’s secondary objectives are to:

- Identify key features (*e.g.,* patient characteristics, clinical markers, biomarkers, *etc.*) that distinguish between RAUC-eligible and -noneligible patients and are predictive of the outcomes, based on the model’s representations (*i.e.,* explainability).
- Prepare baseline models for future work:
  - Predicting the time to surgery for RAUC-eligible patients (*i.e.,* the time interval between ED admission and operating theatre entry), as a means of prioritizing urgent cases.
  - Predicting the hospital LOS for these patients, which can guide bed management and postoperative care provision.
  - Predicting 30-day unplanned readmission after the emergency surgery and 30-day postoperative mortality, to enable risk stratification for enhanced postoperative monitoring.
- Train and internally validate predictive models (*i.e.,* incorporating both structured data fields and unstructured clinical text) for each of the above tasks, and to evaluate their performance and clinical significance.

Our overall goal is to integrate these models (once validated) into a digital triage tool for optimizing patient inclusion and management in the RAUC pathway and, ultimately, improving outcomes (e.g., reducing the 30-day hospital readmission rate).

## Methods

### Study design and setting

**Fig 2:**
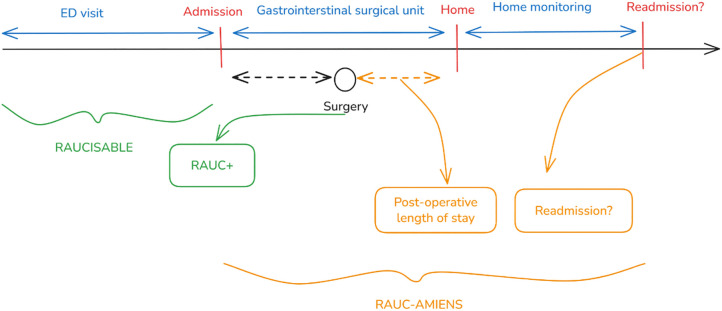
The study timeline, according to the SPIRIT guidelines [18].

RAUCisable has been designed as a single-centre, retrospective, observational cohort study of electronic health record data. The setting is the ED and the Gastrointestinal Surgery Department at the Amiens-Picardie University Hospital (APUH), a tertiary academic medical centre in Amiens, France. APUH coordinates the RAUC project and serves a large territory, with approximately 60,000 visit s to the adult ED per year. The study will use data from a four-year period (from January 1^st^, 2021, to December 31^st^, 2024), in order to ensure that the sample is large enough and to capture any seasonal or annual time trends. No prospective enrolment and no interventions are planned; RAUCisable is a retrospective study of routine care data. The study protocol was approved for implementation on January 17^th^, 2025. The data extraction and curation were initiated thereafter; only after completion of these preparatory steps will the model development and analysis be initiated. The analysis is expected to span 2026, with production of the results by December 2026 (Fig 2).

### Eligibility criteria and cohort definition

#### Inclusion criteria.

All adult patients having attended the APUH ED between January 1^st^, 2021, and December 31^st^, 2024, will be screened. The primary outcome label for model development is RAUC + , which is defined as an adult patient with an index ED visit, subsequent admission for gastrointestinal surgery, and the performance of emergency gastrointestinal surgery within 72 hours of the index ED visit (*i.e.,* by analogy with the RAUC-AMIENS trial’s inclusion criteria). Encounters not meeting all of these criteria will be labelled as “RAUC-”. Emergency gastrointestinal surgery is defined as any urgent surgical intervention on the gastrointestinal tract (*e.g.*, appendectomy, cholecystectomy, bowel obstruction surgery, perforation repair, *etc.*) performed because of an acute condition (i.e., as opposed to elective or scheduled surgery). In practice, these patients are typically admitted via the ED and taken to the operating theatre within hours or days of admission. We shall use admission records and financial logs to track admissions. Thus, the RAUCisable cohort will effectively consist of all ED patients during the study period, with a subclass identified as “RAUC+” (*i.e.,* those who underwent emergency gastrointestinal surgery). The classification models will be developed to detect this class.

#### Exclusion criteria.

Encounters involving direct transfer from the ED to the operating room or direct admission to the ICU will not be included in the primary modelling cohort. This choice reflects the model’s intended use (*i.e.*, to facilitate the early identification of potentially eligible patients in standard ED pathways. These situations might correspond to a distinct subset of patients who are often recognized clinically at a very early stage, and their inclusion might (at least in part) shift the prediction target away from the present study’s main objective. Patients who have formally objected to the research use of their personal health data will also be excluded. There are no exclusion criteria related to incomplete datasets at this stage; however, missing data will be accounted for during the analysis.

### Data sources and data extraction

This study will leverage two institutional primary data sources: the ED information system and the hospital inpatient database. At APUH, the ED information system (ResUrgences, Berger-Levrault, Boulogne-Billancourt, France) records detailed data on each emergency visit, including timestamps (*e.g.,* for triage time, physician evaluation, and disposition), presenting complaints, vital signs, triage category, clinical notes, diagnoses, procedures in the ED, and outcome (*e.g.,* whether admitted, discharged, transferred, *etc.*)). The hospital inpatient database (DxCare, Dedalus, Florence, Italy) contains information on all hospital stays, including admission and discharge dates, surgical procedures (with procedure and diagnosis codes), laboratory and imaging results, and progress notes from inpatient care. Data on outpatient consultations (pre-hospital or as part of the follow-up) can also be accessed but the core focus is on data from the ED and subsequent emergency admissions.

For RAUCisable, we shall extract data from each system and then merge them into a single dataset for analysis. In practical terms, all ED visits in the date range will be extracted from ResUrgences with a unique patient identifier and visit identifier that can be cross-referenced against the inpatient records. The visits that resulted in a admission to a surgical ward will be linked to the corresponding inpatient stay record, in order to retrieve surgical details and outcomes. The data will be extracted by the APUH’s clinical data warehouse team and study data managers, using structured query language (SQL) scripts and/or the hospital’s health data hub facilities. To ensure the comprehensive capture of relevant information, a data extraction plan specifying each variable has been developed.

The structured data fields to be extracted include demographics (age and sex), mode of arrival, triage level (emergency severity index or the equivalent), initial vital signs, presenting symptom codes, comorbidities (*e.g.,* chronic illnesses, medications, and possibly the medical history or medication lists), blood test results on admission, imaging findings (*e.g.,* presence of free air on abdominal X-rays, as available in radiology reports or coded results), time intervals (*e.g.,* the time from ED arrival to the decision for surgery and the time from decision to knife-to-skin), and outcome data (*e.g.,* date of surgery, post-operative complications during the stay, LOS in days, discharge disposition, 30-day readmission status, and 30-day mortality).

Unstructured data will include triage nurse notes, ED physician notes, surgical reports, and discharge summaries. Using natural language processing, additional features that might not be fully captured in structured fields will be extracted.

All extracted data (Table 1) will initially reside within the hospital’s secure environment for cleaning and preprocessing. A study database will be built. Each row will represents an ED visit (the index visit) and will be enriched with fields from the hospital stay, if applicable. Each visit record will have a label indicating whether it was “RAUC+” (*i.e.*, emergency surgery performed) or not, according to the criteria given above. This labelled dataset will form the basis for model training. For the primary classification model, only variables available at triage or during the initial ED assessment will be eligible as predictors. Variables that become available later in the ED stay or after the decision for surgery will not be used as predictors for this primary task and will be reserved for outcome ascertainment or secondary analyses.

**Table 1:** List of variables. LOS, length of stay; ED, emergency department; ICU, intensive care unit; APUH, Amiens-Picardie University Hospital; CCMU, French Clinical Classification of Emergency Patients; ICD-10, International Classification of Diseases, 10th Revision.

Data	Extraction and transformation	Used as a predictor for RAUC+	Used as a predictor for post-operative LOS
First (given) name and last (family) name	Used in preprocessing at APUH to delete all occurrences of the patient’s name on any documents and free text. This field will be deleted from the dataset before transfer to the MatriCS computing platform		
Stay number	Transformation by stay number, with storage of the correspondence table at APUH		
Permanent patient identifier	Transformation by stay number, with storage of the correspondence table at APUH		
Timestamp of arrival in the ED	Day of the week, week of the year, time of arrival	PREDICTOR	PREDICTOR
Sex	Male/female	PREDICTOR	PREDICTOR
Birthday to age	Birthday, transformed into age	PREDICTOR	PREDICTOR
Address	Transcoded into home-APUH distance and travelling time	PREDICTOR	PREDICTOR
**Triage stage**			
Triage priority	The FRENCH scale [19]	PREDICTOR	PREDICTOR
Reason for attendance	The *Société Française de Médecine d’Urgence* classification [20]	PREDICTOR	PREDICTOR
Type of arrival	Own vehicle, ambulance, or mobile ICU	PREDICTOR	PREDICTOR
Referrer	Patient, general practitioner, or emergency medical service	PREDICTOR	PREDICTOR
Waiting position	Standing, sitting, or lying on a stretcher	PREDICTOR	PREDICTOR
Family	No family, family not informed, or family informed	PREDICTOR	PREDICTOR
Triage nurse notes	Free text, pseudonymized	PREDICTOR	PREDICTOR
**Medical status**			
Medical observation	Free text, pseudonymized	PREDICTOR	PREDICTOR
Other specialty observation	Free text, pseudonymized	PREDICTOR	PREDICTOR
Medical history	Free text, pseudonymized	PREDICTOR	PREDICTOR
**Vital signs: time series**			
Heart rate	Beats per min	PREDICTOR	PREDICTOR
Respiratory rate	Cycles per min	PREDICTOR	PREDICTOR
Glasgow	Glasgow coma scale	PREDICTOR	PREDICTOR
Capillary glycaemia	mmol/L	PREDICTOR	PREDICTOR
Oxygen saturation	Percentage	PREDICTOR	PREDICTOR
Oxygen flow	L/min	PREDICTOR	PREDICTOR
Body temperature	°C	PREDICTOR	PREDICTOR
Capillary haemoglobin level	g/dL	PREDICTOR	PREDICTOR
**Medications: time series**			
Drug	International name, administration route, quantity	PREDICTOR	PREDICTOR
**Blood tests: time series**			
Molecule or ion	Name, amount	PREDICTOR	PREDICTOR
**Radiological examination of the abdomen: time series**			
Conclusions	Free text pseudonymized	PREDICTOR	PREDICTOR
Raw images	DICOM	PREDICTOR	PREDICTOR
**Discharge**			
Diagnosis	ICD-10	PREDICTOR	PREDICTOR
CCMU grade	1-5	PREDICTOR	PREDICTOR
Orientation	Admission (to a hospital ward or unit) or discharge		PREDICTOR
Time of operating theatre entry	Day of week, hour, and time interval since ED arrival		PREDICTOR
Time of the end of the patient pathway	Day of week, hour, and time interval since ED arrival		**TARGET**
Post-surgical consultation	Time series: day of week, hour, and time since ED arrival		
Emergency readmission	Time series: day of week, hour, and time since ED arrival, ICD-10		
Surgical unit readmission	Time series: day of week, hour, and time since ED arrival, ICD-10		
Date of death	Day of the week, hour, and time since ED arrival		
RAUCISABLE	Calculated variable: **RAUC + , if the patient is an adult presenting at the ED, subsequently admitted for emergency gastrointestinal surgery, and undergoing emergency gastrointestinal surgery within 72 hours of the index ED visit. Encounters not meeting these criteria are labelled as RAUC-.**	**TARGET**	

### Data processing and pseudonymization

All analyses will be conducted using the statistical software packages in Python 3: scikit-learn [21], pandas [22], keras [23]. The MatriCS platform provides a computing environment suitable for machine learning tasks on large datasets.

#### Pseudonymization and confidentiality.

In compliance with data protection regulations and to protect patient privacy, all data will be pseudonymized prior to analysis outside the hospital’s secure network. Each patient’s identifying information (*e.g.,* name, surname, exact date of birth, address, *etc.*) will be stripped out of the dataset. Instead of real identifiers, we shall use a coded unique study ID for each patient: the permanent patient identifier (PPI). The mapping between patient identities (*i.e.,* hospital IDs) and PPIs will be maintained in a separate encrypted file at the hospital and will not be accessible to the analysis team on external platforms. The patient’s unique hospital identifier will be transformed using a combination of a one-way cryptographic hash function (SHA-256) and a secret salt stored at the hospital. For example, a patient with a “123456789” PPI might be hashed (with a secret salt) into an irreversible but reproducible code like “43a3d35bc82f…908cf6”. A patient who is readmitted will have the same PPI and will not be considered to be a new patient. This ensures that no directly identifiable information is present in the analysis dataset but allows potential linkage back to the source records by authorized personnel, if needed for validation. Free-text documents will automatically scanned for typical name patterns; using an internal dictionary, any occurrences of patient names, addresses, or contact details in the clinical notes will be redacted. Thus, the dataset for analysis will not contain any direct identifiers.

#### Transfer to a high-performance computing platform.

All pseudonymized data intended for model training will be transferred via an encrypted channel (SSH) to the MatriCS secure computing platform (Jules Verne University of Picardie, Amiens, France) for analysis. The data archive will be encrypted with strong (GPG RSA 4096-bit) encryption. Only authorized study investigators will have access to data on the analysis platform, and even that dataset will be a pseudonymized version. The original data will remain on the hospital’s servers and will be accessible only to personnel bound by a duty of medical confidentiality. If the source data have to be checked (*i.e.,* for quality control or audit purposes), the correspondence table (*i.e.,* the correspondence between the study ID and the PPI) will be used within the hospital by authorized staff and will never leave the secure hospital environment. All study personnel with data access are trained in patient confidentiality and have a duty of confidentiality.

#### Data processing.

Given the retrospective nature of the study, data quality and preparation are critical steps. To handle missing values, outliers, and inconsistencies, we shall clean the data. We do not plan to delete visits that have some missing data; indeed, data not purposely collected might be missing at random [24]. Continuous variables (*e.g.,* vital signs and lab test results) will be checked for physiologically implausible values and extreme outliers; these values may be omitted if they are clearly erroneous and will replaced by a null value. Categorical variables (*e.g.*, triage categories and diagnosis codes) will be standardized (*e.g.,* one-hot-encoded). If certain key variables have a high missingness rate, appropriate imputation methods will be used (*e.g.,* multiple imputation or the use of “unknown” categories for categorical data) and carefully documented in all derived publications. We shall also create new derived variables (*i.e.,* indicating the missingness status of the value) that may improve prediction.

The dataset is organized at the visit level, meaning that each variable is specific for the index visit. Subsequent events (*e.g.,* 30-day readmission and 30-day mortality) are identified using the PPI and expressed as time intervals relative to the index visit, or coded as missing when no subsequent event is found in the database. There will not be any data leakage between the training set and the test sets.

#### Natural language processing (NLP).

To extract useful features, free-text clinical notes will be analyzed using NLP techniques. Prior to analysis, these texts will be pseudonymized, and any personal identifiers will be removed. Once the text has been de-identified, we plan to use text mining to identify keywords or concepts indicative of severity (*e.g.*, “sepsis”, “rigid abdomen”, “shock”, etc.) or specific diagnoses. We might employ algorithms such as spaCy or similar NLP libraries to parse the text, or a bag-of-words/embedding approach to incorporate text data into the model. One approach will involve the generation of binary features for the presence of certain terms (*e.g.,* a predefined lexicon of important words related to emergency abdominal conditions) and possibly the use of word embedding or topic modelling to capture more complex patterns in the narratives. These text-derived features will complement the structured data in the predictive models.

#### Outcomes and definitions.

Since this is an observational, data-centred study, our “outcomes” correspond to both the events we are seeking to predict and our predictive models’ performance metrics. The key outcomes of interest are as follows:

- RAUC pathway eligibility (the primary classification outcome): this is a binary outcome indicating whether a given index ED visit meets the RAUC+ rule, i.e., ED attendance, subsequent admission to hospital for gastrointestinal surgery, and emergency gastrointestinal surgery within 72 hours. It serves as the ground truth for training the classification model that will distinguish RAUC-eligible patients from other patients. In the context of the RAUCisable study, this outcome is determined retrospectively for each case on the basis of whether emergency surgery was performed.
- Time to surgery: a continuous outcome measured in hours. For RAUC-eligible patients, this is defined as the time interval between ED admission (*i.e.,* the triage timestamp) and the surgical procedure’s start time. For patients having undergone two or more emergency operations, only the earliest will be considered. This outcome will be used to train a regression model (or possibly a survival model) for predicting how quickly surgery is needed. In practice, we might also categorize this outcome into clinically relevant strata (*e.g.,* surgery within 6 hours, within 12 hours, or within 24 hours) for decision-making, in line with the RAUC triage post-operative categories suggested (<12 h *vs.* < 24 h *vs.* longer). However, to retain granularity, the model will probably treat this prediction as a continuous variable.
- LOS: a continuous outcome defined as the total length (in days) of the hospital stay during which the emergency surgery took place (i.e., from admission or surgery to hospital discharge). We shall predict LOS as a proxy marker of resource utilization and recovery speed. If a patient is transferred to another facility, LOS will be censored at transfer. Patients who die during the hospital stay will have LOS recorded up to the time of death, and death will be accounted for separately as the mortality outcome.
- 30-day readmission: a binary outcome indicating whether the patient was unexpectedly readmitted to any acute care hospital within 30 days of discharge from the index hospital stay (*i.e.,* the stay with emergency surgery). This outcome will be determined by reviewing the hospital’s records and (potentially) any linked regional health system data. However, since this is a single-centre study, we shall capture readmissions to our hospital and assume that most patients return to the same hospital if issues arise; we note this as a study limitation. Unscheduled readmissions will be a primary measure of the RAUC programme’s success. The prediction of the readmission risk will help us to identify patients who might benefit from closer post-discharge follow-up (*e.g.,* telehealth check-ups).
- 30-day mortality: a binary outcome indicating whether the patient died within 30 days of the emergency surgery. This includes in-hospital death during the initial stay or any post-discharge death within 30 days. The outcome will be determined from hospital records and by checking the civil vital status registry, if available: in France, hospitals are allowed to query civil registries for death notifications. This outcome will be relatively rare but is crucial for the identification of high-risk patients.

In addition to these patient-centred outcomes, an important study output will be the set of the predictive models’ performance metrics. The primary performance measure for classification will be the area under the receiver operating characteristic curve (AUROC), along with the sensitivity, specificity, and positive predictive value at chosen probability thresholds. For regression outcomes (*i.e.,* time to surgery and LOS), we shall evaluate metrics such as the mean squared error. For binary outcomes like readmission and mortality, we shall probably also use classification models (*e.g.,* the AUROC). Furthermore, we shall compute measures of feature importance from the models (*e.g.*, using permutation importance or Shapley additive explanation (SHAP) values to identify the variables that contribute most strongly to predictions. These importance rankings serve as secondary outcomes and might highlight key, clinically informative, predictive factors.

Overall, the RAUCisable study’s success will be measured by our ability to develop models with good discriminant ability (*e.g.*, an AUROC >0.8 for the primary classifications) and to generate clinically interpretable insights that feed into the design of prospective RAUC interventions.

### Statistical analysis and model development

All two-sided statistical tests in descriptive comparisons will use a significance level of α = 0.05. However, given that our primary objective is prediction rather than hypothesis testing, p-values will be interpreted cautiously in the context of large samples in which even small differences become “significant”. The present study is a prediction study rather than a comparative causal analysis; hence, methods intended to reduce treatment-selection bias (such as propensity score matching) will not be part of the analytical strategy.

#### Sample size considerations.

During the 4-year study window, we expect to observe between 240,000 and 300,000 visits to the APUH adult ED (i.e., 60,000–75,000 visits per year). This very large sample provides high statistical power for detecting even faint patterns and is particularly advantageous for training data-intensive machine learning models. We expect the number of RAUC-eligible patients (*i.e.,* for emergency gastrointestinal surgery) to be substantially smaller; based on hospital records, this will be a few hundred per year. For example, if ~2% of ED visits culminate in emergency gastrointestinal surgery, there will be 2,000–3,000 RAUC-eligible cases over 4 years; this is an estimate, and exact numbers will be determined from the data. This number is sufficient to train robust, predictive models – in fact, one reason for using the full 4-year span is to ensure that there are enough outcome events, especially for rarer outcomes like 30-day mortality that might account for a few percent of surgical patients). Conventional sample size “power” calculations are less applicable here because we are building predictive models and not testing a specific hypothesis via inference. Nonetheless, the large N will allow us to (i) split data into ample training and validation sets and (ii) include a high-dimensional feature space (with many variables) without overfitting because the rule-of-thumb of requiring a certain number of events per predictor will be satisfied. In summary, the dataset size is determined by data availability and the need for comprehensive machine learning training and is sufficiently large to meet the study’s objectives.

#### Analysis overview.

The analysis will proceed in four main steps: descriptive statistics, predictive model development, model performance evaluation, and explainability.

**Descriptive statistics:** We shall first characterize the overall ED cohort and the subset of RAUC-eligible surgical patients. Continuous variables will be summarized as the mean ± standard deviation or the median [interquartile range], depending on distribution. Categorical variables will be summarized as the count (percentage). We shall compare the RAUC-eligible group with other groups, in order to highlight differences (using t-tests for continuous variables and χ² tests for categorical variables). Key baseline characteristics to be reported include age distribution, sex, common presenting diagnoses, vital sign abnormalities, proportion admitted, *etc.* For the RAUC-eligible group specifically, we shall detail the indication for surgery (*e.g.,* appendicitis, perforation, *etc.*), the surgical procedures performed, the median operating time, complication rates, mean LOS, and readmission and mortality rates. This provides context and might also guide feature selection (*e.g.,* if certain variables differ greatly from one group to another). We shall also evaluate the completeness of data and any patterns of missingness by applying imputation or sensitivity analyses, if needed.

**Predictive model development:** For the primary classification task (*i.e.,* identifying RAUC-eligible patients at the ED triage stage), we shall use supervised machine learning classification techniques. Potential algorithms include logistic regression (with L1/L2 regularization, if needed), random forests, gradient boosted trees (*e.g.,* XGBoost or LightGBM), and (if appropriate) modern methods like deep learning (although interpretability will always be considered). We shall probably begin with simpler, interpretable models (logistic regression) and then move to more complex models if they offer performance gains. Feature selection could be performed through a combination of expert knowledge (including variables known to be relevant to surgical decision-making) and algorithmic methods (like tree-based feature importance or recursive feature elimination on the training set). We shall use k-fold cross-validation on the training data to tune model hyper-parameters and prevent overfitting. The dataset will be split chronologically or randomly into a training set (e.g., 80% of encounters) and an independent hold-out validation set (20%) for the evaluation of final performance. To simulate prospective performance in the latest year, we might prefer a chronological split: for instance, the use of 2021–2023 data for training and 2024 data for validation.

For continuous outcome predictions (*e.g.,* time to surgery and LOS), we shall use regression models. We might opt for algorithms like linear regression after log-transforming skewed outcomes like LOS, or regression variants of the above machine learning methods (*e.g.,* random forest and regression). Alternatively, survival analysis techniques (like Cox proportional hazards models and survival forests) might be used for time-to-surgery if we consider patients who did not undergo surgery immediately as being censored beyond a certain timeframe. However, given that every RAUC-eligible patient ultimately undergoes surgery (by definition) we shall know the actual times, so standard regression suffices.

For binary outcomes (*e.g.,* readmission and mortality), we shall again use classification models. These could potentially be combined with the primary classification: for instance, a multi-output model that first identifies surgical patients and simultaneously predicts their risk outcomes. However, it may be more straightforward to first identify the surgical cohort and then develop separate predictive models within that cohort for readmission and mortality risks, using the data available at end of their initial hospital stay or (if we aim to predict early) even data available on admission).

Throughout the model development phase, we shall incorporate the unstructured text features (extracted via NLP) alongside the structured data. For example, we might include indicators like “peritonitis_mentioned=Yes/No” as a feature. If advanced NLP (*e.g.,* word embeddings) is used, we could integrate these features into a deep learning model. Given the complexity, an iterative approach will be used to ensure that the text data adds value to the structured data.

To avoid time leakage, predictor availability will be anchored to predefined clinical timepoints. The primary classification model will use only information available up to triage completion or the first clinical assessment in the ED. Variables collected later (including laboratory and imaging results obtained after triage, specialty consultation notes, operative reports, discharge summaries, and disposition variables) will not be used as predictors for the primary endpoint.

**Assessment of the model’s performance:** We shall evaluate the final model’s level of performance on the hold-out validation set or, if appropriate, via the cross-validation results. For the RAUC eligibility classifier, we shall plot the receiver operating characteristic (ROC) curve and report the AUROC. We shall also select a probability threshold that might be used in practice (perhaps one that is sensitive enough to capture all cases of surgery, while maintaining reasonable specificity) and report the confusion matrix at that threshold (*i.e.,* true positives, false negatives, *etc.*), as well as precision and recall. For regression outcomes, we shall report the mean squared error and mean absolute error on validation data, and possibly R-squared for the variance explained. In clinical time-to-event predictions, R² might be low; in that case, we shall focus on the magnitude of the error. For binary outcomes like readmission, we shall again use the AUROC or (perhaps) precision-recall curve for class imbalance.

We shall employ techniques to guard against overfitting – notably by keeping the test set fully separate and by using cross-validation. For ease of implementation in the future, we might also consider simplifying the models if the difference in performance is small.

To assess any observed difference in performance between models, the latter will be trained on several random seeds applied to a dataset split and on the weight initiations, and compared statistically using the Wilcoxon signed-rank test [25].

**Explainability of Artificial Intelligence:** Lastly, we shall examine the feature importance in the best-performing model. For tree-based models, feature importance scores (*e.g.,* Gini importance or permutation importance) are readily available. For logistic regression, we shall look at the predictor’s odds ratio and thus identify the most predictive factors. These findings will be used to shape the RAUC pathway (*e.g.*, the refinement of inclusion criteria, or targeted interventions for high-risk features).

We shall perform post-hoc explainable AI analyses on the best-performing models. Overall interpretability will be addressed using partial dependence plots (PDP) to visualise the marginal effect of individual predictors on the model’s predictions across the dataset. For local interpretability and case-level explanations of model output, we shall apply SHAP and local interpretable model-agnostic explanations (LIME). SHAP values will be computed for each prediction, in order to attribute the contribution of each feature and thus offer a unified measure of feature importance across instances. LIME will be used to approximate the local behaviour of complex models (*e.g.,* tree ensembles) by fitting an interpretable surrogate model around a given prediction; this should help clinicians to understand why a model produces a given risk score for a given patient. These techniques will be particularly useful for identifying potential biases, validating clinical plausibility, and facilitating trust in the model when deployed in clinical environments.

#### Study timeline.

The overall timeline for RAUCisable is as follows (see also Fig 2):

- Data extraction: this began in January 2026, immediately after the end of the inclusion period (2024). This phase includes querying the ED and hospital databases for all required data and building the merged dataset. The expected duration is ~ 2–3 months (by February 2026), including data cleaning.
- Data processing and management: during and after extraction, roughly 3 more months (up until June 2026) will be needed for pseudonymization, NLP feature extraction, and the preparation of analysis files.
- Analysis: model development and statistical analysis will take approximately 6–8 months (from June to December 2026). This includes iterative model training, validation, and writing up the results. Given the volume of data, substantial computing time will be allocated on the MatriCS platform.
- Study completion: the planned study end date is December 2026, by which time a final study report will have been prepared. We anticipate having preliminary results earlier (in the autumn of 2026) to guide the design of the RAUC-ALGO trial.
- Dissemination: manuscripts (including the current protocol article and subsequent articles on results) and conference presentations will follow. The RAUCisable results will also be shared with the RAUC project steering committee, for integration into the RAUC-AMIENS and RAUC-ALGO trial logistics.

No interim analyses are planned because this is not an interventional trial and all the data must be collected prior to analysis. However, if data extraction reveals unexpected and significantly different numbers or major data issues, the plan may be adjusted (*e.g.*, extension of the extraction to early June 2026 to increase the sample size or the refine inclusion criteria).

#### Ethical and regulatory considerations.

This study is subject to the French legislation on noninterventional, retrospective observational analyses of existing health data. Accordingly, the RAUCisable study did not require the provision of formal written informed consent from each patient; instead, it falls under the “Reference Methodology 004” (MR004) provisions of the French National Data Protection Commission (*Commission Nationale de l’Informatique et des Libertés*, Paris, France) for the research use of routinely collected health data. The APUH’s registered, overarching commitment to MR004 (registration number 2208336, dated 9 Oct 2018) covers the RAUCisable study. In compliance with MR004 requirements, patients whose data are included have been informed of the study through public notices and have been given the opportunity to opt out. Specifically, information about RAUCisable, its objectives, procedures and datasets was made available via the hospital website and posters in the hospital, and patients could exercise their right to refuse the use of their data and thus be excluded from the study.

The study protocol was nevertheless submitted to the local independent ethics committee for confirmation that it meets the criteria for non-interventional research (reference: PI2025_843_0015) and has been registered at ClinicalTrials.Gov (NCT07037719) [26].

#### Dissemination plan.

The results of the RAUCisable study will be disseminated through several channels. We plan to publish the main findings in a peer-reviewed journal, with a focus on the predictive models’ development and performance. Furthermore, insights into key predictive features will be shared with the clinical community (possibly in a surgery or emergency medicine conference), to inform clinicians about any important risk factors identified. Importantly, the RAUCisable outcomes will feed directly into the design of the RAUC-ALGO trial; for example, the algorithmic trigger for pathway activation in RAUC-ALGO will be based on RAUCisable’s model. We shall include our algorithm (or a simplified score derived from it) in the RAUC-ALGO intervention. The RAUCisable findings will also be disseminated to relevant APUH departments (*i.e.,* the ED, surgical wards, the anaesthesia unit, and quality improvement) because they highlight the hospital’s current performance and areas for improvement in emergency surgical care (*e.g.,* the typical time interval to surgery and its impact). There is also potential to share data or collaborate with other centres (*i.e.,* RAUC-OUVERT will externally validate the approach at another hospital), and an aggregated multicentre analysis might follow. All individual-level data will remain secure; only de-identified, aggregated data or possibly a trained model (which does not contain patient data) would ever be shared beyond the institution.

## Results (anticipated)

As this is a protocol paper, no final results are available. Below, however, we outline the expected outcomes and how they will be reported upon completion of the study:

### The study population

We expect to identify ~2,400 RAUC-eligible patients from among *~*250,000–300,000 ED visits over 4 years. These patients are likely to be significantly older than non-surgical ED patients (*e.g.*, with many over the age of 65), with a higher proportion of acute abdominal conditions (*e.g.,* ~ 24% with appendicitis, ~ 13% with bowel obstruction, ~ 10% with peritonitis, *etc.*), and higher initial disease acuity on triage. We shall probably find that for RAUC-eligible patients, the 30-day readmission rate is substantial (probably ~20–30%, based on the literature and local data) and the 30-day mortality rate is perhaps around 5–10% (reflecting disease severity). The median time interval between ED entry to surgery might be around 8–12 hours, although we expect the range to be broad. The median LOS might be ~ 7 days but this will depend on the procedure and the presence or absence of complications.

### The primary outcome

We expect to see a strongly discriminant machine learning model for identifying RAUC-eligible cases at triage. Our preliminary experience suggests that variables like extreme heart rate, hypotension, very elevated inflammatory markers, or keywords indicating surgical abdomen will be telling features. The target performance is an AUROC of ≥0.90 for this classification, which will effectively create an AI-based triage that matches or exceeds the accuracy of clinician-based triage. To illustrate the net benefit of using the model, we shall present a ROC curve and, potentially, a decision curve analysis.

### Secondary outcomes

We expect to identify clinically intuitive but important key predictors: for example, age, the American Society of Anesthesiologists score, the comorbidity burden, the initial blood lactate level, signs of peritonitis on examination (from text data), and need for vasopressors in the ED might emerge as strong predictors of 30-day mortality. Similarly, a prolonged ED dwell time and a lack of early surgery might correlate with worse outcomes and underscore the need to expedite care; this evidence would be supportive of RAUC’s goals. For readmissions, surgical findings (such as the presence of stoma, which associated with readmission according to the literature [3]) and social support might be highlighted.

Prediction of the time to surgery: we expect to achieve moderate success: factors such as presentation time (day vs night), severity (shock prompting earlier surgery), and operating theatre availability and scheduling might all have an influence. For example, the model might predict whether or not a patient will be in the operating theatre within 6 hours of ED entry with reasonable accuracy (perhaps an AUROC of 0.8). Continuous prediction of times might have an error margin of a few hours. To align with the RAUC planning, we shall probably categorize predicted surgery as urgent (<12 h), semi-urgent (12–24 h), and delayed.

Prediction of LOS: the prediction model might highlight patients likely to stay for a very long time, due to complications. We expect that certain features (*e.g.*, the presence of sepsis or the need for ICU admission) will predict prolonged stays. While the exact LOS day by day is hard to predict, identifying the top decile (*i.e.,* very long stays) could be feasible with reasonable accuracy.

We anticipate being able to predict readmissions with an AUROC of ~0.80–0.85, i.e., similar to studies that used electronic health record data [27]). Key predictors might include complications during the index stay, socioeconomic factors (if available in the data), and lack of early follow-up. The mortality prediction models might also have an AUROC of ~0.85, given that mortality is often foreseeable with combinations of age, comorbidities, and acute conditions.

It is important to note that these anticipated results are based on our hypotheses and a review of similar studies; the actual findings will be reported in detail once the analysis has been completed.

## Discussion

To the best of our knowledge, RAUCisable will be one of the first studies to apply advanced data analytics and machine learning to care pathways for patients requiring emergency gastrointestinal surgery [28–30]. By retrospectively examining all emergency presentations over several years, we are seeking to derive actionable insights that can improve patient outcomes in future prospective trials and in routine clinical practice. Below, we discuss the potential implications of the RAUCisable study, its strengths and limitations, and how it integrates into the larger RAUC programme and the growing field of digital health in surgery.

Integrating AI with clinical pathways: a key innovative feature of RAUCisable is its integration of AI-driven prediction into a clinical decision pathway. Importantly, the model is intended to be an early screening aid and not an automated indication for surgery. A positive prediction should be interpreted as prompting expedited clinical review, repetition of the abdominal examination, and accelerated diagnostic work-up when appropriate. Conventionally, identifying patients for an ERAS programme or a clinical trial is a manual process based on inclusion criteria. The RAUCisable model’s intended role is to facilitate the early recognition of potentially eligible patients within normal ED workflows, rather than to replace the clinical identification of cases requiring immediate escalation. For example, if our algorithm can flag up a patient as likely to need emergency surgery within hours, the necessary resources (*e.g.,* an operating theatre, an ICU bed, *etc.*) can be prepared, and elements of the RAUC protocol (*i.e.,* goal-directed resuscitation, minimizing fasting time, *etc.*) can be initiated promptly. This proactive approach aligns with the broader goal of a predetermined patient trajectory using AI-based triage, as envisioned in the RAUC project. It demonstrates how digital tools can augment clinical decision-making but helping clinicians to make data-informed decisions rapidly, rather than replacing clinicians. As studies in emergency medicine have shown, machine learning can enhance triage accuracy and predict patient disposition or outcomes effectively [17]; ultimately, these tools can increase throughput and improve care quality.

### Impact on the RAUC-AMIENS trial

The RAUCisable study’s findings will directly guide the implementation of the concomitant, prospective, controlled RAUC-AMIENS trial of the augmented recovery pathway (including ERAS elements and remote monitoring). Firstly, RAUCisable will help to characterize the target population and baseline care trajectories. Secondly, RAUCisable might identify certain high-risk subgroups (*e.g.,* patients above a certain age with sepsis are extremely likely to benefit). The RAUC-AMIENS could consider pre-specified subgroup analyses or even focused adaptive enrolment of these groups. Thirdly, the baseline outcome rates (*i.e.,* readmission and complication rates) that we will measure retrospectively will serve as historical controls or as a context for RAUC-AMIENS to demonstrate improvement. Moreover, RAUCisable’s data-driven identification of patients ensures that RAUC-AMIENS can better target eligible patients in real time – potentially by using a simplified version of our model to screen admissions during the trial recruitment phase. In this way, the retrospective study and prospective trial form a feedback loop: retrospective data informs trial design, and trial outcomes will later be used to refine predictive models further as more data becomes available.

### Generalization and the RAUC-OUVERT study

We acknowledge that RAUCisable is a single-centre study, which limits generalizability. Practice patterns (*e.g.,* criteria for going to the operating theatre, and typical time intervals) and patient demographics at our institution may differ from those at other institutions. To mitigate this, the RAUC-OUVERT study will test the RAUC pathway (and by extension, our triage algorithm) in another hospital to provide external validation. We anticipate that while certain specifics may differ (*e.g.,* another hospital might have shorter time intervals or a different case mix), the core predictors of bad outcomes (*e.g.,* physiological impairments) are universal. Nevertheless, site-specific quirks might make the model’s level of performance fall when applied elsewhere. Therefore, one of the long-term goals is to make the model more robust via continuous learning and updating with multicentre data.

### Strengths, limitations, and future work

Strengths: RAUCisable has a large sample with rich real-world data, which increases the reliability of any patterns found. The inclusion of unstructured data via NLP is a strength because a lot of critical information in emergency cases is documented in narrative form (*e.g.,* “rebound tenderness” noted by a physician is highly predictive of surgical abdomen). By capturing this information, we go beyond the many predictive models that rely on coded data only. Furthermore, the tight link to an ongoing clinical programme (RHU RAUC) means that our study is poised for translation into practice; this is not just a theoretical prediction exercise but corresponds to the materialization of a triage tool tested in RAUC-ALGO. The study also rigorously addresses data security and ethics and shows that this type of retrospective, big-data research can comply with patient privacy requirements – an increasingly important consideration in the era of health informatics.

The RAUCisable study also has several limitations. Firstly, as a retrospective study, we depend on the accuracy and completeness of the recorded data. Missing data or documentation errors (*e.g.,* a miscoded diagnosis or failure to record vital signs) could affect the model training process. We have plans for imputation and will conduct sensitivity analyses to see whether or not the exclusion of cases with missing critical fields changes the results. Secondly, outcomes like readmission might be underestimated because we only capture readmissions to our hospital; our data cannot tell us whether a patient went to a different hospital. However, this is an inherent limitation of single-centre studies. We shall acknowledge this limitation and might use proxy measures (*e.g.,* if the patient was seen in our ED for a complication but was not admitted). France has a national health system database (Système National des Données de Santé (SNDS)) that can track patients from one hospital to another; in future work, we could link to SNDS for more complete outcome capture. However, regulatory constraints mean that this beyond the scope of the current protocol. Thirdly, our predictive model (regardless of how good it might be) captures correlation and not causation. If the model predicts higher mortality for patients with certain features, these features will not inevitably cause death – in fact, they are severity markers. The model should be used to support decision-making and not to dictate decisions; clinicians will always need to apply their own judgment. In the RAUC-ALGO trial, we shall have to be careful to use the algorithm in a way that complements clinical assessment (*e.g.,* providing risk scores to the surgeons who make the final decision). The risk of over-reliance on AI or algorithmic bias (*e.g.,* if the data had biases in treatment given to some groups) is real. We plan to examine the model’s fairness by (for example) ensuring it works equally well in older patients vs younger patients, in males vs females, *etc.*, and does not inadvertently reinforce disparities. Fourthly, encounters involving direct transfer to the operating room or ICU are not included in the primary modelling cohort. These cases might represent a particularly severe subset of surgical emergencies. This choice was made to maintain consistency with the model’s intended clinical use in routine ED pathways, although it may somewhat limit the applicability of the model to the most immediately apparent surgical presentations. Lastly, a surgery-based outcome label does not fully capture the clinical severity on presentation. Some high-risk abdominal patients might require close monitoring and repeated examinations without ultimately undergoing surgery, whereas others might only become apparent later in the ED stay. These clinically important situations might appear as false positives or false negatives, relative to the RAUC+ label. We therefore view false positives as being clinically informative (rather than purely erroneous) and consider them to be relevant for the subsequent development of hierarchical or multiclass triage models.

#### Future directions.

If RAUCisable’s models prove to be accurate, the next step (RAUC-ALGO) will implement them prospectively. In the RAUC-ALGO trial, we shall be able to measure whether or not using the algorithm-driven triage actually changes outcomes (*e.g.,* lowers complication rates or speeds up care), compared with the standard of care. Success in the RAUC-ALGO trial might pave the way for the wider integratation of AI tools into EDs. Furthermore, the combination of telemedicine/e-health with the identification of high-risk patients could ensure that the latter are closely followed up after discharge, in order to potentially preventing readmissions. In essence, RAUCisable provides the “who” and “what to expect”, RAUC-AMIENS provides the “how to provide care,” and RAUC-ALGO will test the combination of “who and how” in improving outcomes.

## Conclusion

We believe that the RAUCisable study will be a pivotal step toward digitally enhanced emergency surgical care. By learning from past data, we are seeking to improve the future management of emergency surgery patients through timely identification and the provision of targeted care pathways. This protocol paper has detailed our methodological approach for ensuring rigor and reproducibility. We anticipate that RAUCisable will yield valuable models and knowledge, such as a model for the early detection of surgical emergencies and risk stratification tools for outcomes such as readmission. These deliverables will directly inform and enhance the broader RAUC initiative and will ultimately contributing to lower complication and unplanned readmission rates and higher survival rates for patients who undergo emergency gastrointestinal surgery. We believe that by combining classical clinical insights and modern AI techniques, this approach exemplifies the future of acute care research and quality improvement. If successful, RAUCisable could serve as a model for integrating AI into clinical trial design and emergency care pathways in other settings and thus marking a significant advancement in emergency surgery management.

## Supporting information

S1 File RAUCisable SPIRIT 2025 editable checklist. (DOCX)

S2 File RAUCisable protocol auto translated EN. (PDF)
