# Supplementary material for: Identification and prediction of patients eligible for augmented rehabilitation in emergency gastrointestinal surgery (RAUCisable): A protocol for a single-centre, retrospective, observational study
Source: PLoS One. 2026 Jun 3;21(6):e0350113. doi: 10.1371/journal.pone.0350113 (PMC13232787; doi:10.1371/journal.pone.0350113)
Supplement: S2 File — (PDF) [file pone.0350113.s002.pdf]

# RESEARCH PROTOCOL NOT INVOLVING HUMAN PERSONS

Identification and prediction of patients eligible for RAUC

Single-center study    Study acronym: RAUCisable

Registration number: PI2025_843_0015

Study version: No. 1.18 - 17/03/2025

**Coordinating investigator:**

Professor: REGIMBEAU Jean-Marc    Department: Visceral Surgery

Department address: CHU Amiens-Picardie

80054 Amiens Cedex FRANCE    Tel: 03.22.08.80.00.

Email: regimbeau.jean-marc@chu-amiens.fr

DRCI reference: PI2025_843_0015
RAUCisable - Version No. 1.18 - 17/03/2025

DOCUMENT AUTOMATICALLY TRANSLATED

# TABLE OF CONTENTS

DESCRIPTION OF THE RESEARCH
RIGHTS OF ACCESS TO DATA, CONFIDENTIALITY, DESCRIPTION OF DATA AND SOURCE DOCUMENTS
ACCESS TO DATA
DATA CONFIDENTIALITY
DETAILED DESCRIPTION OF THE DATA COLLECTED
SOURCE DATA
QUALITY CONTROL AND ASSURANCE
INSTRUCTIONS FOR DATA COLLECTION
RESEARCH MONITORING
AUDIT AND INSPECTION
ETHICAL AND REGULATORY CONSIDERATIONS
GENERAL PRINCIPLES
PROTECTION OF PERSONS
AMENDMENT TO THE PROTOCOL
PROCESSING, RETENTION AND ARCHIVING OF DOCUMENTS AND DATA RELATING TO THE RESEARCH
JUSTIFICATION FOR DATA COLLECTION
DATA FLOW AND SECURITY
DATA PROCESSING, VERIFICATION AND VALIDATION METHODS
DATA RETENTION
DATA ARCHIVING
RULES RELATING TO PUBLICATION
SCIENTIFIC COMMUNICATIONS
COMMUNICATION OF RESULTS TO PATIENTS
TRANSFER OF DATA
BIBLIOGRAPHICAL REFERENCES
APPENDICES

# LIST OF ABBREVIATIONS

| ABBREVIATION | DEFINITION OF THE TERM |
|---|---|
| **IOA** | Reception Organizing Nurse |
| **SFMU** | French Society of Emergency Medicine |
| **AP** | Private ambulance |
| **VSAV** | Emergency and victim-assistance vehicle |
| **SMUR** | Mobile Emergency and Resuscitation Service |
| **MT** | General practitioner / treating physician |
| **C15** | Centre 15 |
| **ATCD** | Medical history / antecedents |

# 1. DESCRIPTION OF THE RESEARCH

| | |
|---|---|
| **DATA CONTROLLER** | CHU Amiens-Picardie |
| **COORDINATING INVESTIGATOR** | Prof. Jean-Marc REGIMBEAU |
| **TITLE** | Identification and prediction of patients eligible for RAUC (RAUCisable) |
| **RATIONALE / BACKGROUND** | Identifying patients to be included in RAUCAMIENS can be difficult in the emergency department (patient flow, night shifts, residents/senior physicians from other specialties covering shifts). This preliminary study therefore aims, on the one hand, to optimize inclusion of patients in the forthcoming pivotal study (detection of RAUC+ patients), on the other hand to identify variables of interest (variable importance), and finally to pre-train models predicting time to operating room and length of stay (study on the RAUC+ subgroup). |
| **OBJECTIVES** | Identify patients who may be eligible for inclusion in the RAUC AMIENS research protocol.<br>Identify the relevant data for classifying patients.<br>Predict time before operating room.<br>Predict the length of hospital stay of the RAUCisable subgroup.<br>Predict readmissions based on emergency-department data from RAUCisable patients.<br>Predict death at Day 30. |
| **RESEARCH DESIGN** | Single-center, retrospective, observational study using data only. |
| **INCLUSION CRITERIA** | All adults consulting the emergency department of CHU Amiens-Picardie. |
| **NON-INCLUSION CRITERIA** | Patients refusing access to their data.<br>Patients objecting to the use of their data for research purposes. |
| **RESEARCH TREATMENTS / STRATEGIES / PROCEDURES** | Extraction of data from ResUrgences + hospital stays (and consultations). Creation of a single dataset. RAUCisable labeling by a clinical research technician via a script. Model training on MatriCS. Performance measurement. |
| **OUTCOME MEASURES** | ROC curve of classification models.<br>Mean squared error of regression models.<br>Variable importance. |

DOCUMENT AUTOMATICALLY TRANSLATED

| | |
|---|---|
| **STUDY POPULATION SIZE** | 2021-2024 x 60,000 visits/year = 300,000 patients.<br>This patient volume is necessary to allow model training on all columns taken into account. |
| **PLANNED NUMBER OF CENTERS** | One inclusion center only: CHU-AP (processing: CHU-AP and MatriCS (UPJV)). |
| **TIMELINE** | Extraction boundaries: 01/01/2021 - 31/12/2024. Research start date: 17/01/2025. Data collection / entry / management: 6 months.<br>Analysis of results and model training: 1 year. Total study duration: 1 year.<br>Final report submission date: 31/12/2025. |
| **STATISTICAL ANALYSIS OF DATA** | Population description.<br>Numbers and proportions for categorical variables. Mean and standard deviation for continuous variables.<br>Comparability of imputation methods: t-test of the squared error. |
| **EXPECTED IMPACT** | Model for detecting includable patients.<br>Model predicting time to surgery for RAUCisable patients.<br>Model predicting length of stay for RAUCisable patients. |

DOCUMENT AUTOMATICALLY TRANSLATED

# 2. RIGHTS OF ACCESS TO DATA, CONFIDENTIALITY, DESCRIPTION OF DATA AND SOURCE DOCUMENTS

## 2.1. ACCESS TO DATA

The manager and the principal investigator are responsible for obtaining the agreement of all parties involved in the research in order to guarantee direct access to all research sites, source data, source documents and reports for quality control and audit purposes.

The persons who direct, conduct and monitor the research will collect the documents and individual data strictly necessary for research monitoring, quality control and audit, and make them available to persons with access to these documents in accordance with applicable legislative and regulatory provisions.

## 2.2. DATA CONFIDENTIALITY

In accordance with the legislative provisions in force, persons with direct access to the source data shall take all necessary precautions to ensure the confidentiality of information relating to the persons concerned, in particular with regard to their identity and to the results obtained. These persons, like the persons who direct and monitor the research, are bound by professional secrecy.

During the research or after its completion, research data collected from the persons concerned and transmitted to the research manager by the persons who direct and monitor the research (or any other specialized contributors) will be coded/pseudonymized. Under no circumstances will they show the names of the persons concerned, their patient identifier, or their address (no direct identification). Pseudonymization will therefore be carried out as follows: hashing of the patient identification number with the addition of a constant character string (commonly called the "salt", which will be stored on the CHU-AP side), using the following algorithm: SHA-256. To illustrate the process, inclusion of a patient with IPP 123456789 and the salt "-sel" would be encoded as:

"43a3d35bc82fbccb54e778ddeef97e0d783d9b6ae753146a24d3b3ccd1908cf6". If a return to the source data is required to ensure data quality and completeness, a correspondence table linking the patient pseudonym to the real IPP will have to be generated; however, access to this table will be: 1/ possible only in the inclusion center, so that no real identity is communicated outside CHU Amiens-Picardie; 2/ optimally secured according to the medium (computerized table encrypted by the principal investigator and located on a secure CHU Amiens-Picardie server); 3/ restricted to the CHU Amiens-Picardie professional(s) involved in the research and authorized under the regulations.

Certain sensitive and/or identifying data will undergo specific pseudonymization processing on the CHU-AP side (see data flow) before the final processing of these data on the MatriCS side:

Pseudonymization of free-text fields and documents:

The patient identity will be used within the CHU-AP environment to remove all occurrences from free-text fields and replace them with the tag "[PATIENT_IDENTITY]".

Pseudonymization of the remaining text will follow the ETALAB guide: https://guides.etalab.gouv.fr/pseudonymisation

Text anonymization:

Stemming: removal of suffixes (e.g., abdominal and abdomen will be transformed into abdom; pain, painful [masculine/feminine] will be transformed into pain).

Tokenization: each stemmed word will be represented by a code.

Vectorization: representation of these words in a dimensional space allowing concepts to be brought closer together in a geometric space.

Similarly, in order to comply with the principle of minimization, post-emergency-department data from RAUC- patients will be removed from the dataset transmitted to MatriCS.

## 2.3. DETAILED DESCRIPTION OF THE DATA COLLECTED

All data collected are relevant, scientifically justifiable, and limited to what is necessary with regard to the objectives pursued by the study.

In addition, all data collected fall within the categories of data authorized by CNIL Reference Methodology No. 4.

The list below exhaustively sets out all data collected for the research:

| DATA | DEFINITION AND METHODS USED |
|---|---|
| **Patient surname and first name** | Used during the preprocessing phase on the CHU-AP side to remove any occurrence of the patient identity from all documents and free-text fields. This data item will be deleted before transfer to MatriCS. |
| **Stay number** | Correspondence table on the CHU side |
| **Date / time of emergency-department admission** | Day of the week, week of the year, rounded hour |
| **Sex** | Male or female |
| **Age** | Date of birth transformed into age (in months) |
| **Address** | Transcoded into time and distance from CHU-AP |
| **IOA** | IOA |
| **IOA prioritization** | FRENCH |
| **Reason for arrival** | SFMU reason for arrival (catalogue) |
| **Mode of arrival** | Self-presenting, private ambulance, VSAV, SMUR |
| **Referral mode** | Spontaneous, general practitioner, Centre 15, transfer |
| **Waiting modality** | Ambulatory, chair, stretcher |
| **Family** | Notified, in the waiting room, non-existent |
| **IOA observation** | Free text pseudonymized by tokenization, stemming and vectorization |
| DATA | DEFINITION AND METHODS USED |
| **Medical observation** | Free text pseudonymized by tokenization, stemming and vectorization |
| **Specialist-opinion observation** | Free text pseudonymized by tokenization, stemming and vectorization |
| **Medical history** | Free text pseudonymized by tokenization, stemming and vectorization |
| **Vital signs: time series** | Vital signs: time series |
| **Heart rate** | In bpm |
| **Respiratory rate** | In cycles per minute |
| **Glasgow Coma Scale** | According to the ad hoc scale from 3 to 15 |
| **Capillary blood glucose** | In mmol per liter |
| **Oxygen saturation** | In % |
| **Oxygen flow rate** | In liter(s) per minute |
| **Temperature** | Degrees C |
| **Capillary hemoglobin** | g/dL |
| **Prescription: time series** | Prescription: time series |
| **Medication** | Name of the medication (or INN), route of administration, dosage, date of administration |
| **Biological assessment** | Biological assessment |
| **Na, K, ...** | Name of the assay, assay value, assay date |
| **Abdominal imaging of the patient** | Abdominal imaging of the patient |
| **Text report** | Free text pseudonymized by tokenization, stemming and vectorization |
| **Raw images** | Linked to the stay number: DICOM or JPEG format |
| **Patient orientation** | Patient orientation |
| **Diagnosis** | ICD-10 |
| **CCMU** | 1-5 |
| **Discharge orientation** | Hospitalization (and in which department) or discharge |
| **Date and time of operating room** | Day of the week and rounded hour, and exact time to operating room |
| **Date of discharge from surgery** | Day of the week and rounded hour, and length of stay |
| **Outpatient consultations in visceral surgery** | Time series of delays relative to the inclusion date |
| **Emergency-department readmission** | Time series of delays relative to the inclusion date + ICD-10 |
| **Rehospitalization in visceral surgery** | Time series of delays relative to the inclusion date + ICD-10 |
| DATA | DEFINITION AND METHODS USED |
| **Date of death** | Time between the date of surgery and death, where applicable |
| **RAUCISABLE** | This variable will be calculated using the following algorithm: RAUC+ if an adult patient is hospitalized for visceral surgery after passing through the emergency department and has an operating-room visit within 72 hours; RAUC- otherwise, or in the event of surgical revision (prior surgery), departure to the operating room before admission to visceral surgery, pregnancy or breastfeeding. The quality of this algorithm will be assessed by sampling with visceral surgeons and may be optimized. |

DOCUMENT AUTOMATICALLY TRANSLATED

## 2.4. SOURCE DATA

Any original document or object that makes it possible to prove the existence or accuracy of a datum or fact recorded during the research is defined as a source document.

These documents are: ResUrgences + hospital-stay database + DxCare file.

In accordance with CNIL Reference Methodology No. 4, all data relating to the persons concerned come from the data subjects themselves (or their legal representatives) and/or from the professionals involved in the research (e.g., patient records) and/or from legally constituted databases or biological-sample collections that have undergone the necessary formalities with the competent authorities. It should be noted that these sources may cover data collected outside CHU Amiens-Picardie.

# 3. QUALITY CONTROL AND ASSURANCE

## 3.1. INSTRUCTIONS FOR DATA COLLECTION

All information required by the protocol will be entered directly in pseudonymized form (see section 2.2) into one or more CSV files located exclusively in the Research Space (see section 5) after pseudonymization processing. Data will be collected as they are obtained and transcribed clearly.

A scripted automatic extraction of data from the source documents to the CSV file will be carried out by the investigators or authorized persons.

The data will be processed as entered in the computerized patient record, with their errors and imperfections, except for the target variables (raucisable, hospitalization, time to operating room and length of stay).

## 3.2. RESEARCH MONITORING

Research monitoring will be ensured by the principal medical investigator. He will be responsible for directing the research and ensuring:

- the logistics and monitoring of the research;

- the preparation of reports on its progress;

- verification that the case report form is up to date (requests for additional information, corrections, etc.).

He will work in accordance with standardized operating procedures.

## 3.3. AUDIT AND INSPECTION

An audit may be carried out at any time by persons mandated by the research manager and independent of the persons responsible for the research. Its objective is to ensure the quality of the research, the validity of its results, and compliance with the applicable laws and regulations.

The persons who direct and monitor the research agree to comply with the requirements of the sponsor and the competent authority regarding an audit or inspection of the research.

The audit may apply to all stages of the research, from protocol development to publication of the results and filing of the data used or produced within the framework of the research.

# 4. ETHICAL AND REGULATORY CONSIDERATIONS

## 4.1. GENERAL PRINCIPLES

The manager and the person(s) who direct and monitor the research undertake to ensure that this research is conducted in compliance with the Declaration of Helsinki (the full version of which can be found at https://www.wma.net/what-we-do/medical-ethics/declaration-of-helsinki/).

The data recorded for this research are subject to computerized processing at CHU Amiens-Picardie in compliance with Law No. 78-17 of 6 January 1978 on information technology, files and freedoms, as amended by Law 2004-801 of 6 August 2004.

This research falls within the framework of CNIL "Reference Methodology No. 4" (MR004). CHU Amiens-Picardie has signed a commitment to comply with this methodology (No. 2208336 dated 9 October 2018). This research is therefore recorded in the institution internal register under the number shown on pages 1 and 3 of this protocol.

## 4.2. PROTECTION OF PERSONS

This retrospective study does not involve human persons and therefore does not fall within the scope of the Jarde law, because all data to be collected for the purposes of this research come from data already collected as part of the usual care of patients.

Each person participating in this research will be able to access an information notice (Appendix No. 1 for the patient and Appendix No. 2 for the professional), which will be available on the CHU-AP website. This document will inform them of:

- the identity of the data controller and of its Data Protection Officer;

- the nature and purpose of the data collected;

- the recipients of the data;

- the retention and archiving period of their data;

DOCUMENT AUTOMATICALLY TRANSLATED

- the possibility of stopping the study at any time and of the retention, by the manager, of the information collected;

- their rights of access, objection, rectification, restriction and erasure regarding the data collected. These rights may be exercised at any time, either by submitting a request to the investigator or to the Data Protection Officer designated by the research manager;

- the possibility of lodging a complaint with the CNIL.

The research manager undertakes, through the investigators or the Data Protection Officer, to respond to any request for objection, access, rectification, restriction or erasure of data within a maximum period of one month from receipt of the request.

### 4.3. AMENDMENT TO THE PROTOCOL

Any substantial modification shall be the subject of a written amendment submitted to the research manager and to the Methodology and Data Management Center, where applicable.

All protocol amendments must be brought to the attention of all healthcare professionals participating in the research, who undertake to comply with their content.

## 5. PROCESSING, RETENTION AND ARCHIVING OF DOCUMENTS AND DATA RELATING TO THE RESEARCH

### 5.1. JUSTIFICATION FOR DATA COLLECTION

The data collected for this research are collected for scientific purposes, in the public interest with regard to its aims, the improvement of medical knowledge targeted by the topic addressed, and its purpose of making the results obtained public (oral or written communication, etc.).

### 5.2. DATA FLOW AND SECURITY

An authorized investigator mandated by CHU Amiens-Picardie will extract the data from the databases, then pseudonymize them on the CHU-AP RAUC server (see section 2.2 for the pseudonymization procedure). The CHU-AP RAUC server will be operated by the Digital Services Department (DSN) of CHU Amiens-Picardie. Operational access (to the data and to perform processing) to this server will be restricted to investigators authorized and mandated by the principal investigator, and will be through a personal identifier and personal password provided by the DSN of CHU Amiens-Picardie to the coordinating investigator, as well as to authorized and mandated collaborators requiring access (see Appendix No. 3). The server will be backed up daily. Before transfer to the MatriCS RAUC server, the data will be encrypted, signed and archived in the Research Space of the DSN of CHU Amiens-Picardie, which will allow logging of this process (traceability of access and modifications related to the data). Directly identifying data will never be entered in the Research Space and will remain on the CHU-AP RAUC server, access to which will be restricted to legally authorized local persons.

The data will be transferred to the MatriCS platform for the calculation of the models targeted by the primary and secondary objectives. This transfer will take place after pseudonymization, via an archive encrypted with a GPG RSA 4096 certificate and signed by the GPG RSA 4096 certificate of the person responsible for the transfer, and via an SSH channel to a closed and dedicated area of the MatriCS platform (MatriCS RAUC server).

From the point of view of overall information-system security, the internal network of CHU Amiens-Picardie is secured by a firewall that protects the CHU network from any external intrusion. A proxy server also controls internet browsing, and antivirus software examines all files and pages copied from external servers to CHU Amiens-Picardie. Anyone wishing to connect to the CHU network must first identify themselves using a username and password provided by the CHU IT services.

### 5.3. DATA PROCESSING, VERIFICATION AND VALIDATION METHODS

Data management of the pseudonymized database (see section 2.2) located in the Research Space will be carried out upstream of the statistical analysis in a succinct and automatic manner for relative range checks (data to be verified), absolute range checks (outlier data), and missing data (accepted because the raw data are relevant). This step will be carried out by Dr. Emilien ARNAUD. Depending on the specifications, data validation may be carried out for the statistical analysis, and correction requests may be sent to the investigator in order to complete and correct the data. The database lock/unlock process must be decided between the person responsible for performing the statistics, the person responsible for managing the database, the coordinating investigator and any collaborators.

### 5.4. DATA RETENTION

The data collected in the context of this research will be retained for a period of 2 years from the start date of collection in the inclusion center, but will be deleted from the computing platform as soon as the calculations are no longer necessary. In all cases, the data collected may not be retained beyond two years after the last publication of the research results. In the absence of publication, they may be retained only until submission of the final research report.

### 5.5. DATA ARCHIVING

At the end of the retention period, the data will be electronically archived for a maximum duration of 20 years or for a duration compliant with the regulations in force.

## 6. RULES RELATING TO PUBLICATION

### 6.1. SCIENTIFIC COMMUNICATIONS

DOCUMENT AUTOMATICALLY TRANSLATED

Analysis of the study data is carried out by Emilien ARNAUD and Momar DIOUF using Python 3.11 software. This analysis gives rise to a written report submitted to the research manager. This report enables preparation of one or more publications.

Any written or oral communication of the research results must receive the prior agreement of the principal investigator and, where applicable, of any committee set up for the research.

In accordance with the provisions of the French Data Protection Act, presentation of the results shall in no way allow the direct or indirect identification of the persons concerned by the research.

Publication of the main results mentions the name of the sponsor, all investigators who included or followed patients in the research and, where applicable, the methodologists or biostatisticians who participated in the research, members of the committee(s) set up for the research, and the funding source. International rules for authorship and publication will be taken into account (Vancouver Convention, February 2006).

### 6.2. COMMUNICATION OF RESULTS TO PATIENTS

At their request, patients are informed of the overall results of the research.

### 6.3. TRANSFER OF DATA

No transfer of data is planned for this protocol.

## 7. BIBLIOGRAPHICAL REFERENCES

NA

DOCUMENT AUTOMATICALLY TRANSLATED
